# Progression of an Invasive ACTH Pituitary Macroadenoma with Cushing's Disease to Pituitary Carcinoma

**DOI:** 10.1155/2015/810367

**Published:** 2015-08-20

**Authors:** Clarissa Groberio Borba, Rafael Loch Batista, Nina Rosa de Castro Musolino, Vanielle Carvalho Machado, Ana Elisa Evangelista Alcantara, Gilberto Ochman da Silva, Valter Angelo Sperling Cescato, Malebranche Berardo Carneiro da Cunha Neto

**Affiliations:** ^1^Neuroendocrinology Unit, Functional Neurosurgery Division, Hospital das Clinicas, Faculdade de Medicina, Universidade de São Paulo, Brazil; ^2^Pituitary Neurosurgery Unit, Functional Neurosurgery Division, Hospital das Clinicas, Faculdade de Medicina, Universidade de São Paulo, Brazil

## Abstract

Pituitary carcinomas are very rare tumors that in most cases produce prolactin and adrenocorticotropic hormone (ACTH). It is a challenge to diagnosis of a pituitary carcinoma before disclosed symptomatic metastasis. We report the case of a female patient with Cushing's disease who underwent three transsphenoidal surgeries, with pathological findings of common ACTH pituitary adenoma including Ki-67 expression <3%. She achieved hypocortisolism after the 3rd surgery although ACTH levels remained slightly elevated. The patient returned some time later with fast worsening of hypercortisolism. Magnetic resonance imaging showed clivus invasion, which led to a fourth surgery and radiation. This time, immunohistochemistry revealed strong Ki-67 (10% to 15%) and p53 expression. Liver and lumbar spine metastases were found on workup. The patient died after few months due to lung infection. Pituitary carcinomas are rare, and the transformation of an ACTH-secreting pituitary adenoma into a carcinoma is exceptional. The difficulty of defining markers for the diagnosis of carcinoma, before metastasis diagnosis, in order to change the management of the disease, is a challenge.

## 1. Introduction

Pituitary adenomas are a common type of intracranial tumor. The more effective approach is transsphenoidal surgery. Nonfunctioning pituitary adenomas and prolactinomas are more prevalent pituitary adenomas [1]. Pituitary adenomas can be classified according to pathological, radiological, or clinical behavior as typical or atypical, invasive or noninvasive, and aggressive or nonaggressive. The World Health Organization (WHO) classification categorizes pituitary adenomas as typical and atypical and the pathological features of atypical adenoma are defined as a Ki-67 index greater than 3% and/or extensive p53 immunoreactivity [1]. Pituitary carcinomas are very rare (0.1%-0.2% of pituitary tumors) [2], with diagnosis requiring evidence of metastasis and they, frequently, show a higher index of Ki-67 and p53 protein than aggressive adenomas. Most of these tumors secrete prolactin and/or ACTH and they are usually resistant to radiotheraphy [1, 3, 4].

## 2. Case Description

**Figure 1:**
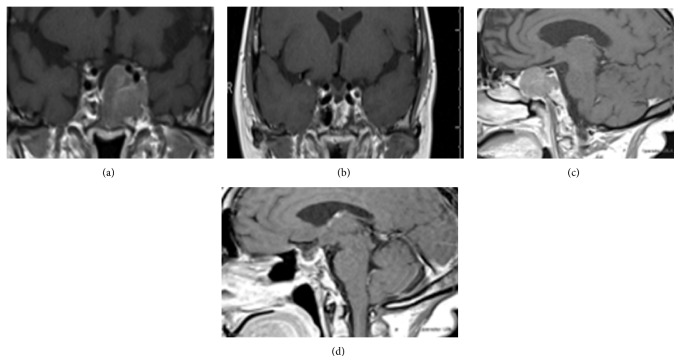
(a) Coronal image before the third surgery. (b) Sagittal image before the third surgery. (c) Coronal image after the third surgery. (d) Sagittal image after the third surgery.

**Figure 2:**
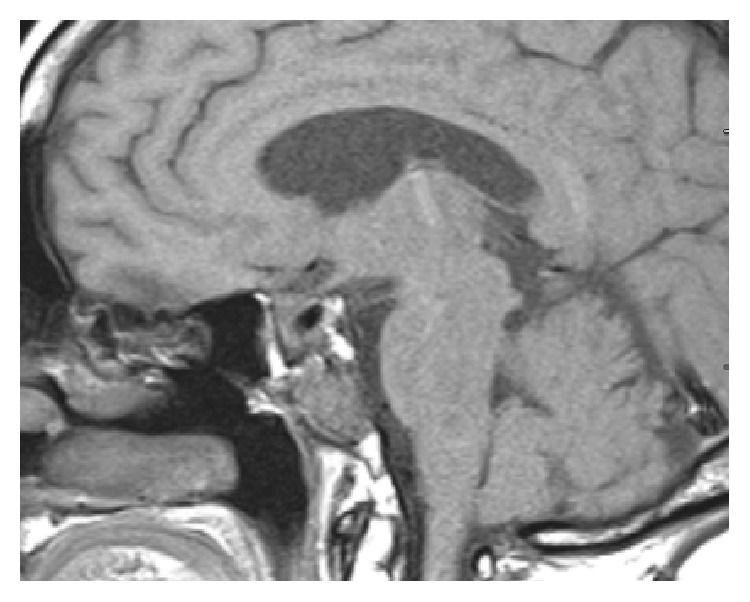
Sagittal image on clinical recurrence showing tumor invasion of the clivus.

**Figure 3:**
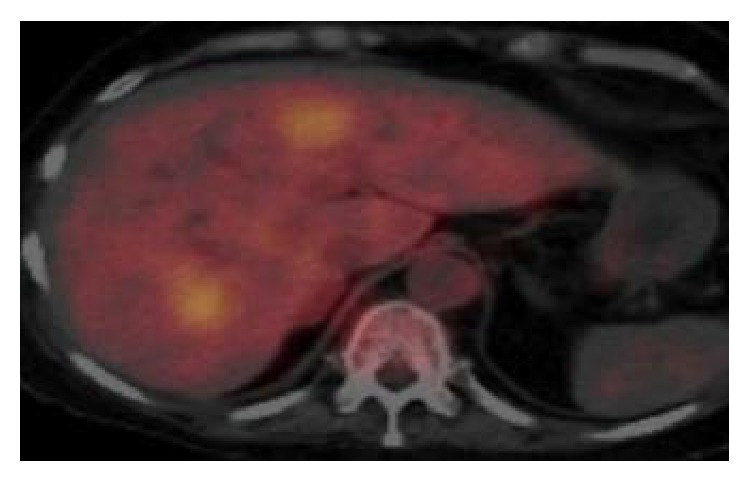
Liver metastasis (PET/CT).

**Figure 4:**
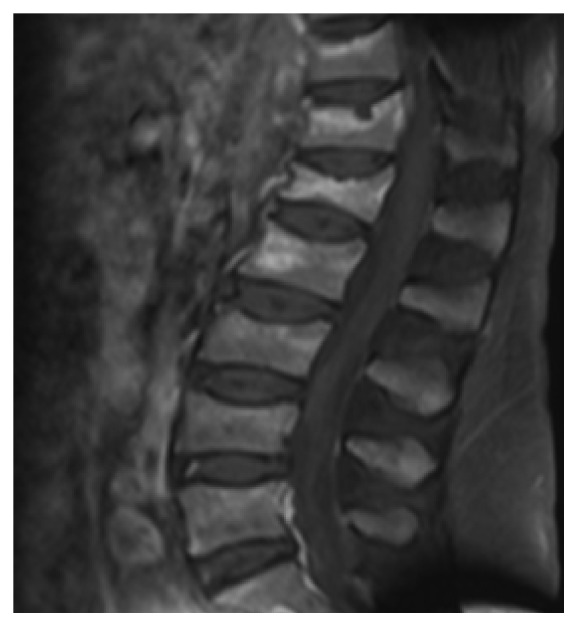
Lumbar spine metastasis on MRI.

A 55-year-old female patient diagnosed with Cushing's disease (CD) due to a macroadenoma (2,39 × 2,54 × 3,36 cm) underwent two transsphenoidal surgeries (TS) at another medical center (2008 and 2009, and we do not have data about the anatomopathological exam of these surgeries because these procedures were done in another region of country), without clinical improvement. She presented to our hospital by the end of 2009 with persistent CD symptoms. The patient did not use any medication when she came to our hospital. Laboratory tests revealed hypokalemia (*K* = 2.4 mEq/L, normal range 3.5–5 mEq/L) and very high serum ACTH and cortisol levels (Table 1). Magnetic resonance imaging (MRI) showed an invasive sellar mass (Figures 1(a) and 1(b)). The patient exhibited symptoms of pituitary apoplexy confirmed after a third TS with radical tumor resection (Figures 1(c) and 1(d)). Histopathology confirmed a pituitary adenoma positive for ACTH (standard diffuse > 50%) and Ki-67 (<5%) and negative for p53 by immunohistochemistry (IHC) (Table 2). The patient achieved complete clinical and laboratory remission of hypercortisolism. In fact, she developed clinical and laboratory adrenal insufficiency requiring glucocorticoid replacement for two years. No tumor growth was detected, although ACTH levels remained slightly elevated (Table 1). In 2012, the patient returned with rapidly progressive recurrence of clinical and laboratory hypercortisolism (Table 1). Sellar MRI showed tumor extension into the clivus (Figure 2). The patient was started on ketoconazole (1200 mg/day) and underwent radiotherapy (5040 cGy in 28 sessions) after another subtotal TS resection. Immunohistochemical analysis after this fourth surgery showed a pituitary adenoma with ACTH expression (>50%) and diffuse immunoreactivity to Ki-67 (10%–15%) and to p53. The patient returned 3 months later, showing accelerated clinical deterioration: hyperpigmentation, severe myopathy and low back pain, dyspnea, generalized edema, severe hypertension, and diabetes mellitus. Laboratory tests showed hypokalemia (2 mEq/L) and very high ACTH and cortisol levels (Table 1). Positron emission tomography (PET/CT) was performed using 10.4 fluoro-2-deoxyglucose mCi labeled with fluorine-18, which showed multiple hypodense nodules in the liver parenchyma, measuring up to 3.3 cm in segment VIII (Figure 3). Liver biopsy showed a neuroendocrine pattern with immunopositive ACTH (>50%) and Ki-67 (5%–10%), Table 1. MRI also showed metastasis in the lumbar spine (Figure 4). The patient was reassigned the oncology team for discussion of the possibility of using temozolomide and adrenalectomy was considered at time. But the patient developed important lung infection, probably due to hypercortisolism, and she died one month later.

## 3. Discussion

Pituitary carcinomas are rare tumors, accounting for an average of 0.2% of pituitary neoplasms. They are defined by the presence of craniospinal and/or systemic metastases and are associated with high mortality rates. Most are invasive sellar tumors, characterized by their expansion beyond the limits of the sella turcica. They show endocrine activity and consist of PRL- and/or ACTH-secreting tumors in most cases. However, these tumors can also be nonfunctioning and produce symptoms only when adjacent structures are compressed [3]. Primary pituitary carcinoma metastases are of difficult diagnosis because they are not always present at the time of initial diagnosis [2, 3]. According to Pernicone et al., the latency period between the diagnosis of a primary carcinoma and the manifestation of metastases ranges between 3 months and 18 years (mean, 6.6 years). This time interval is longer for ACTH-secreting carcinomas than prolactinomas (9.5 versus 4.7 years) [4]. The protein Ki-67 is a cell proliferation marker detected by the monoclonal antibody MIB-1 and is expressed as a percentage of immunopositive nuclei in the form of a Ki-67 proliferation index [5]. According to Thapar et al. Ki-67 over 3% is a criterion to distinguish invasive from noninvasive adenomas with 97% specificity and 73% sensitivity [5, 6]. In this case, after 3 years, the adenoma progressed to a carcinoma, as evidenced by p53 expression and diffuse Ki-67 staining. Typically, pituitary carcinomas develop craniospinal, lung, liver, and ovary metastases. Immunohistochemical staining of these metastases reveals elevated rates of oncoproteins such as p53 and cell proliferation marker (Ki-67), which is helpful for diagnosis and prognostic relevance in these cases [4, 6]. It is worth noting that the levels of those markers are typically higher in metastases than in the primary tumor [6]. The most interesting and unusual aspect in the reported case was that the patient had a diagnosis of an adenoma with low oncoprotein expression and later progressed to a carcinoma with increased oncoprotein expression in the primary tumor and in the liver metastasis, with rapid and severe clinical deterioration—thus demonstrating the malignancy and high mortality of pituitary carcinomas. The treatment options for these tumors are surgery, radiotherapy, and, more recently, chemotherapy with drugs such as temozolomide [7]. The response to these treatment modalities is poor, as 66% of the patients die within 1 year, which illustrates the inexorable course of this serious disease [4].

## 4. Conclusion

Pituitary carcinomas are rare, and the progression of an ACTH-secreting pituitary adenoma to a carcinoma is exceptional. The difficulty of defining before metastasis markers for the diagnosis of carcinoma is a challenge that can be solved by changing the approach to this disease.

## Figures and Tables

**Table 1:** Clinical course.

Data	Condition	24 h urinary cortisol^**∗**^	Late salivary cortisol^**∗∗**^	Serum morning cortisol^**∗∗∗**^	ACTH^**∗∗∗∗**^
Dec 2009	Before 3rd surgery	>1,100	6.45	51.8	793
Dec 2009	**3rd TS surgery**				
Jan 2010	Postoperative (no GC replacement)			4.4	46
Feb 2010	No GC replacement	54.6	0.11	5.7	62
Jun 2010	GC dependency replacement	20	<0.1	2.3	60
Dec 2010	GC dependency replacement	25.2	0.04	3.2	45
Mar 2011	GC dependency replacement	25	0.08	2.8	59
Apr 2012	Clinical recurrence of CS	170	0.12	16.5	270
May 2012	No drug	435	0.21	25.3	336
Jul 2012	On 1,200 mg/d of ketoconazole	821	0.52	23.5	670
Jul 2012	**4th surgery**				
Sep 2012	Postoperative (no drug)	4,998	2.44	47.7	582
Nov 2012	**Radiotherapy**				
Feb 2013	Postradiotherapy	2,348	13.5	70.8	4,087

^*∗*^24h urinary cortisol (NR: 30–310 *μ*g/24 h).

^*∗∗*^Late salivary cortisol (NR < 0.13 *μ*g/dL).

^*∗∗∗*^Serum morning cortisol (NR: 5–25 *μ*g/dL).

^*∗∗∗∗*^ACTH (NR: <46 pg/mL).

GC: glucocorticoid, CS: Cushing's syndrome.

**Table 2:** Tumor markers comparison between third and fourth surgeries.

	Ki-67 (%)	p53	ACTH (IHQ)
2009 (after 3rd surgery)	<5%	No evidence	>50%
2012 (after 4th surgery)	10–15%	25%	>50%

Considerations: The Ki-67 and p53 immunohistochemical exams were made with primary monoclonal antibody performed with 1 : 50 dilution, according to the manufacturer's protocol.
